# The Role of the Microbiota in the Diabetic Peripheral Artery Disease

**DOI:** 10.1155/2019/4128682

**Published:** 2019-05-08

**Authors:** Federico Biscetti, Elisabetta Nardella, Andrea Leonardo Cecchini, Raffaele Landolfi, Andrea Flex

**Affiliations:** ^1^Fondazione Policlinico Universitario A. Gemelli IRCCS, U.O.C. Clinica Medica e Malattie Vascolari, Rome, Italy; ^2^Università Cattolica del Sacro Cuore, Laboratory of Vascular Biology and Genetics, Rome, Italy; ^3^Università Cattolica del Sacro Cuore, Rome, Italy

## Abstract

Vascular complications of diabetes mellitus represent a major public health problem. Although many steps forward have been made to define the causes and to find the best possible therapies, the problem remains crucial. In recent years, more and more evidences have defined a link between microbiota and the initiation, promotion, and evolution of atherosclerotic disease, even in the diabetic scenario. There is an urgency to develop the knowledge of modern medicine about the link between gut microbiota and its host's metabolic pathways, and it would be useful to understand and justify the interindividual diversity of clinical disease presentation of diabetic vascular complication even if an optimization of pharmacological treatment has been made or in the case of young patients where hypertension, dyslipidemia, and diabetes are not able to justify a very quick progress of atherosclerotic process. The aim of the present review is to gather all the best available evidence in this regard and to define a new role of the microbiota in this field, from biomarker to possible therapeutic target.

## 1. Type 2 Diabetes Mellitus: A Chronic Low-Grade Inflammatory Disease

Type 2 diabetes mellitus (T2DM) represents a chronic metabolic disease characterized by a relative insulin deficiency due to pancreatic *β*-cell dysfunction and insulin resistance in target organs, with consequent hyperglycemia. It has become a global public health problem because of an endemic progression worldwide, also resulting from an increasing prevalence of obesity and sedentary lifestyle [1]. Indeed, T2DM is considered as a chronic, low-grade inflammatory disease determined by long-term immune system imbalance, metabolic syndrome, and/or nutrient excess [2].

Emergent evidences support the implication of inflammatory processes with an abnormal production of cytokines and activation of inflammatory signaling pathways in the development of this metabolic disease [3–6]. In the early 1990s, Hotamisligil et al. described an increase of tumor necrosis factor- (TNF-) *α* in adipose tissue and, conversely, an improved peripheral glucose uptake with the neutralization of TNF-*α* in animal models of obesity and diabetes [7, 8]. This finding marked a new era in understanding that a subclinical inflammatory process triggers both insulin resistance and metabolic dysfunction, which precede T2DM. Advances in this field have recognized components of both innate and adaptive immune responses in regulating the inflammatory process [9]. Even, Tsai et al. have hypothesized that T2DM could be considered as an autoimmune disease [10]. In addition, T2DM is clearly associated with macro- and microvascular complications that are considered as the expression of the inflammatory process [11]. In particular, atherosclerosis is a complex process resulting from an inflammatory response to injury with the interaction of numerous cell types and formation of fatty streaks that could progress to atheromatous plaques, plaque destabilization, and plaque rupture [12]. Endothelial dysfunction is an early event of this process that determines the alteration of vascular homeostasis, and it stimulates the production of proinflammatory cytokines [12]. Chronic hyperglycemia condition accelerates the progression of atherosclerosis because of the overproduction of reactive oxygen species (ROS) by the mitochondrial electron transport chain, the formation of intracellular advanced glycation end products, the activation of protein kinase C, and the increase of polyol pathway flux [13]. Excess of ROS also increases the expression of inflammatory and adhesion factors, the formation of oxidized low-density lipoprotein, and insulin resistance by activating the ubiquitin pathway, inhibiting the activation of AMP-protein kinase and adiponectin, and decreasing endothelial nitric oxide synthase activity [12].

### 1.1. Lower Extremity Arterial Disease in Diabetic Patients

Diabetes is associated with accelerated atherosclerotic disease that affects arteries of the brain, heart, and lower extremities [14]. Therefore, diabetic patients have a higher risk of stroke, myocardial infarction, and limb amputation [15]. In particular, peripheral artery disease (PAD), defined as the atherosclerotic occlusive disease of the lower extremities, is one of the most severe conditions in patients with T2DM. Nowadays, PAD represents a public health problem with a significant impact on healthcare and high economic burden [12]. Over 200 million people are affected with lower extremity artery disease worldwide [13], and its prevalence increases with the prevalence of T2DM, one of the major risk factors [16]. Furthermore, PAD has special features and poorer prognosis in diabetic than in nondiabetic patients [17]. Clinical onset is frequently characterized by critical limb ischemia and gangrene, typical manifestations of advanced disease stages, due to a poorly symptomatic progression of these patients during the earlier stage of disease and to their reduced pain perception related to the concomitant presence of peripheral neuropathy [18]. As a consequence, patients with diabetes are at higher risk of lower extremity amputation than those without diabetes [6, 19–21]. In addition, diabetic patients with PAD, compared with diabetic patients without PAD, have also a higher risk of cardiovascular disease [22–25]. Despite its severity, PAD is still the least studied compared to other diabetic vascular complications [26].

## 2. The Microbiota: The Oldest Guest

The human organism owns several metabolic pathways to counter the inflammatory process determined by the continuous exposition to the external environment and pathogens and to endogenous oxidative factors [27]. The infection results as one of both local and systemic principal inflammation-promoting factors [28]. In the latter case, the role of a cross-mimicry process [28–31] and a systemic bloodstream translocation from a local origin [30, 32–34] has been demonstrated as initiating and promoting events of the systemic inflammation burden far from the site of the original colonization or infection [35].

Specifically, several studies have enhanced a clear correlation between *Helicobacter pylori* and atherosclerosis, known as the plaque inflammation process [28, 30, 31, 36]. In addition, genetic fragments of human-colonizing microbes (*Helicobacter pylori* and periodontal microbes) have been found in carotid artery samples [28, 37, 38] of patients affected by PAD, demonstrating an atherosclerotic plaque colonization. Moreover, other bacteria or viruses have been discovered colonizing the atherosclerotic plaques (e.g., *Chryseomonas*, *Veillonella*, *Streptococcus*, *Cytomegalovirus*, human immunodeficiency virus, *Mycobacteria*, *Porphyromonas gingivalis*, *Aggregatibacter actinomycetemcomitans*, *Tannerella forsythia*, *Fusobacterium nucleatum*, and *Streptococcus mutans*) [28, 39–49]. The examples of microorganisms implied in the indirect [30, 40, 41, 50–63] and direct activation of the immunological system determining the plaque atherosclerotic burden are continuously increasing [30, 64–67], confirming the real necessity to deeply understand this topic and so compensate for the actual lack of knowledge about the basic mechanism of the microbiota's role in atherosclerosis [68–71].

Recent studies support the predominant role of the infection at the base of the inflammation load focusing on the outcomes of the actual available therapeutic solutions in lower-limb PAD, such as endovascular revascularization procedures and major vascular surgery. The influence of bacterial activity has been demonstrated in several unfavorable outcomes, such as the restenosis after arterial angioplasty [41, 50, 71–77] or any major adverse cardiovascular event (MACE) [30, 59, 78], which represent the first cause of exitus of patients affected by lower-limb PAD [79]. An interesting possible explanation has been proposed, defining the role of the bacterial atherosclerotic plaque colonization as an additional promoting factor of inflammation burden, after the angioplasty trauma-induced local inflammation [80–83]. The sum of the two stimuli determines the increased production of cytokines, the endothelial dysfunction, the induction of the foam cells, the proliferation and migration of the vascular smooth muscle cells (VSMCs), the powered tendency of platelets to aggregate, and the proinflammatory behavior of the perivascular adipose tissue (PVAT) [30, 40, 41, 51–58, 60, 62, 63, 84–86].

### 2.1. The Microbiome

Recent hypothesis supports a complementary role of microbiota as a constitutive component of the human organism rather than an inevitable and casual colonization from the environment around us. This complementarity has just been introduced and partially understood by the increasing studies that try to highlight the microbiome, the collection of microbial genomes, and the plasticity that completes our genomic feature [64]. The microbiome is the genetic characterization of the entire microbiota in a specific tissue [87]. Its crosstalk with the immune system modulates and regulates the immune response against the host [88]. In particular, the gut microbiome plays a fundamental role in this modulation for its location and microbiota. The gut microbial community is composed mainly of phyla *Bacteroides*, *Firmicutes*, *Actinobacteria*, *Proteobacteria*, and *Verrucomicrobia* [89], in different proportions. Interindividual variation is determined by a difference in the microbiome and also by environmental factors, such as lifestyle, diet, antibiotics, and drug use [90, 91]. This amount of genetic data has been playing an unexplored role in the modulation of our metabolism pathways and in our pathologies, such as obesity [64, 92, 93] and diabetes [64, 94–99]. In the latter case, a different geographic origin influences the gut microbiome showing as similar metagenomes that could encode similar functions presenting a differently marked microbe species composition [64, 99]. The microbiome plasticity is directly influenced by influencing factors of the host itself, such as the intrapartum neonatal colonization through the vaginal canal transit, or totally host-independent factors, such as the change of diet from maternal milk to the introduction of solid food, the level of hygiene to which everyone differently has been exposed since birth, and the use of antibiotic therapy during lifetime [64, 100].

Although the microbiota becomes definitive and adult-like in the host at around three years of age [64, 101], the microbiome still changes through the epigenetic mechanisms that are induced by endogenous and exogenous factors [64].

## 3. The Microbiota and Microbiome in Type 2 Diabetes Mellitus

Evidence in animal and human models supports the hypothesis that obesity and T2DM are associated with a deep gut dysbiosis. Overnutrition could represent one of the main starting points to alter gut microbiota locally and to initiate systemic inflammatory processes through the mucosal barrier [102, 103]. Qin et al. performed the first metagenome-wide association study in T2DM using stool samples from Chinese patients with T2DM [98]. They found that T2DM patients had only a moderate degree gut bacterial dysbiosis. Functional annotation analyses, however, indicated a decline in butyrate-producing *Roseburia intestinalis* and *Faecalibacterium prausnitzii*, which may be metabolically beneficial, and an increase in several opportunistic pathogen levels. Another metagenome-wide association study was performed on T2DM and conducted in Europe on postmenopausal female patients with normal, impaired, or diabetic glucose regulation [99]. In this study, Karlsson et al. found that *Roseburia intestinalis* and *Faecalibacterium prausnitzii* were highly discriminant for T2DM, in contrast to the Chinese cohort. The authors suggest that the two studies were considerably different, not only for the different sequencing techniques used but also for ethnic and dietetic influences. Moreover, a previous smaller study found that T2DM patients showed higher levels of *Lactobacillus* species in comparison to nondiabetics [94], as showed by both Chinese and European studies. In addition, Zhang et al. found that normal subjects differed from patients with prediabetes with higher levels of *Faecalibacterium prausnitzii* and *Haemophilus parainfluenzae* T3T1, whereas *Verrucomicrobiaceae*, *Akkermansia muciniphila*, and *Clostridiales* sp. SS3/4 were less abundant [104]. The last result differs from the findings of Qin et al., which described a reduction of *Akkermansia muciniphila* in Chinese patients with diabetes. These results, however, suggest that patients with T2DM have evidence of gut dysbiosis. The reasons for the discrepancies may be determined by various confounding factors, such as different study populations, different sequencing techniques used, and different diets and drugs used [105].

Recent studies suggest that short-chain fatty acids (SCFAs), such as acetate, butyrate, and propionate, as well as the end products of fermentation of dietary fibers by the anaerobic intestinal microbiota, might constitute a link between the microbiota and systemic inflammatory diseases. In particular, butyrate seems to have a direct role in the development of extrathymic anti-inflammatory regulatory T cells [106]. Trompette et al. demonstrated that mice fed a high-fiber diet have an altered microbiota and are protected from allergic airway inflammation [107]. They showed that propionate regulated allergic inflammation, bone marrow hematopoiesis, and dendritic cell function. These findings suggest that metabolites produced by the gut microbiota influence hematopoiesis and immune responses in the lung. Thus, these microbiota-derived products might be important players in the generation of local and systemic immunity/inflammation. According to the studies mentioned before, the alteration on the production of SCFAs, especially butyrate, observed in T2DM patients, might have a key role in the development of low-grade inflammation [105].

Another important role in the development of a metabolic syndrome has been demonstrated for the pattern recognition receptor such as the toll-like receptor 5 (TLR5), a component of the innate immune system expressed in the gut mucosa and one that helps defend against infection [108]. TLR5-deficient mice exhibited hyperphagia and developed hyperlipidemia, hypertension, insulin resistance, and obesity, as well as an altered microbiota. Interestingly, the transfer of intestinal microbiota from TLR5-deficient mice to germ-free mice led to metabolic syndrome. These data support the crosstalk of gut microbiota with the innate immune system and suggest that the alteration of this link is critical in the development of the metabolic syndrome. In addition, studies show that gut-derived endotoxin—lipopolysaccharide (LPS)—might be involved in the chronic inflammation observed in T2DM. Cani et al. described that a high-fat diet (HFD) increased the LPS content of the gut microbiota and resulted in metabolic endotoxemia [95]. They observed that subcutaneous infusions of LPS into mice determined insulin resistance and obesity similar to that after feeding an HFD. Gut dysbiosis might increase LPS production by gram-negative bacteria and lead to metabolic endotoxemia and low-level inflammation that could contribute to the development of insulin resistance and T2DM [12].

### 3.1. The Microbiota in Atherosclerosis

An evident promoting role of microbes in a nonspecific inflammatory mechanism has been observed supporting an active participation of microbiota in systemic metabolic processes of the human body. At the base of this “inflammasome,” there are several processes, such as an overproduction of proatherogenic mediators (C-reactive protein (CRP); interleukin 18 (IL18), IL1*β*, and IL6; and TNF-*α*), a hyperstimulated expression of adhesive molecules (vascular cell adhesion molecule 1 and intercellular adhesion molecule 1) [28, 30, 40, 41, 50, 85, 109], synthesis and release of growth factors and PVAT-derived adipokines, production of ROS, hormones (corticosteroids and sex hormones), and free fatty acids, and a cytokine-related direct influence on the autonomic nervous system [28, 60, 62, 63]. The latter phenomenon is known as the neuroendocrine-immunitary crosstalk, which finally causes an homeostatic unbalance that initiates and promotes hypertension, insulin resistance, diabetes, altered levels of low-density lipoprotein- (LDL-) cholesterol, plasma triglycerides, high-density lipoprotein- (HDL-) cholesterol [28, 110–112], and a rise of oxidative molecules that determine the LDL-cholesterol oxidation, with a worsening of the atherosclerotic plaque instability and progression [28, 113, 114].

Bacterial colonization/infection of the vascular wall may contribute to the pathogenesis of atherosclerosis by the activation of a local, and eventually systemic, immunological response [115]. This process may involve each of the vascular wall layers (the intima, media, and adventitia) [28]. The main effect of a possible infection on the intima layer is the induction of endothelial dysfunction with a resulting dysregulation in vasomotor function, thrombotic complications, and initiation and progression of atherosclerosis [28]. There are several lines of evidence to suggest that bacterial infection activates platelets by a stimulatory effect on von Willebrand factor binding and factor VIII associated with a hyperfibrinogenemia state [116, 117].

The infection of the media layer may affect VSMC function and connective tissues that participate in the regulation of blood pressure, the vascular lumen, and the modulation of shear stress [84]. The adventitia layer is composed of adventitial compacta and adventitial fat, the aforementioned PVAT [118]. PVAT has recently been defined as the widest endocrine tissue that humans own [62]. It produces adipokines, hormones (corticosteroids and sex hormones), cytokines (TNF-*α*, IL6, and IL8), growth factors (visfatin, platelet-derived growth factor-BB, and transforming growth factor-*β*), and other substances such as ROS, nitric oxide (NO), hydrogen sulfide (H_2_S), free fatty acids, and plasminogen activator inhibitor type 1 [28]. These substances regulate inflammation, vasoreactivity, and vascular VSMC growth, proliferation, and migration in the adjacent layers of the vasculature [84]. Bacterial infection may modify the PVAT functions [28].

An increasing amount of study evidences the association of periodontal bacteria, such as *Porphyromonas gingivalis*, *Tannerella forsythia*, *Prevotella intermedia*, *Aggregatibacter actinomycetemcomitans*, *Treponema denticola*, *Prevotella nigrescens*, *Fusobacterium nucleatum*, *Eikenella corrodens*, *Parvimonas micra*, and *Campylobacter rectu*s, and cardiovascular disease [33, 34, 119]. The study conducted by Tapashetti et al. shows higher CRP plasma level and a greater mean carotid intima-media thickness (c-IMT) value in patients with chronic periodontitis than in patients with healthy gums [119]. Kosaka et al. found that higher levels of salivary inflammatory cytokines were associated with periodontal disease. Among these, higher salivary IL6 and TNF-*α* were positively associated with both periodontal disease and intensity of carotid atherosclerosis [120]. In the case control study conducted by Chen et al., periodontal bacteria was in 13 of the 25 (52%) atherosclerotic samples obtained from patients with aortoiliac and/or femoropopliteal occlusive disease [121]. These results confirm that periodontitis increased fivefold the risk of having PAD and was associated with increased serum IL6 and TNF-*α* concentrations.

New evidences of the importance of microbiota are continuously found in the multifaceted human metabolic network as the proven reduction of the prevalence of *Eubacterium* and *Roseburia* in gut microbiota of patients who have already had an atherosclerotic symptomatic event, with an opposite pattern of prevalence for *Colinsella* [28, 64]. Moreover, a different gut microbiota composition was found in patients affected by diabetes [98] and atherosclerosis, giving the basis to hypothesize an atherosclerotic process induced by a possible gut microbiota dysbiosis [28, 122]. The discovery of the increased dimension and lipid content of the atherosclerotic plaques observed in mice fed with a hyperlipid diet [111, 123] is an interesting example of lipid trim imbalance mediated by the action of colonizing microbes. Chen et al. have demonstrated that levels of *Helicobacter pylori* immunoglobulin G (IgG) and serum IL18 were significantly higher in subjects with increased c-IMT [85]. This evidence suggests a positive association between *Helicobacter pylori* infection and subclinical carotid artery atherosclerosis mediated by IL18. In addition, the coronary atherosclerosis in patients affected by chronic heart disease with an infection of Cag A-positive *Helicobacter pylori* was explained by an infection related to the imbalance between lipid metabolism and LDL-cholesterol oxidation burden, the aggravation of which proved the progression and instability of the atherosclerotic plaque [28, 124]. The importance of this elegant crosstalk between microbiota and the host metabolism is clearly enhanced by a more aggressive disease phenotype observed in a specific group of patients, according to their microbial composition. Indeed, the presence of *Chlamydia pneumoniae* in blood and plaques of these patients has been defined as a promoter of hypercholesterolemia-induced atherosclerosis [111] that could be a possible cause of restenosis after an angioplasty procedure [28, 125, 126]. Another example of microbiotal influence on prognosis could be the evidence of a raised severity of stroke in *Chlamydia pneumoniae* seropositive patients with an increased c-IMT [124, 127, 128].

Several data suggest that infections resulting from periodontal or gut microbes have a direct influence on our endocrine system, on PVAT, and on pituitary-suprarenal action, with a possible derived imbalance of the autonomic sympathetic nervous system and metabolism homeostasis that could induce hypertension, insulin peripheral resistance, T2DM, increase of LDL-cholesterol and triglycerides, decrease of HDL cholesterol associated to an even more oxidative burden by ROS overproduction, and restenosis phenomenon [28, 84]. Moreover, a leaky gut phenomenon that allows a bloodstream translocation of bacterial fragments and a direct atherosclerotic plaque colonization [28, 64] could facilitate several processes, including neuroimmune crosstalk [28, 40, 41, 51–56, 58], macrophage-specific reverse cholesterol transport process modulation [28, 129–131], and the development of many diseases, such as obesity and T2DM [28, 64].

In addition, it was observed that diabetic patients have higher baseline plasma levels of LPS than the healthy control group and a low prevalence of butyrate-producing bacteria (e.g., *Roseburia* and *Faecalibacterium* spp.) known for anti-inflammatory abilities [64, 98, 99].

The atherosclerotic plaque peroxidation is essential for promoting LDL-cholesterol accumulation inside macrophage cells, which become foam cells. These cells promote the upregulation of inflammasomes created by an overproduction of cytokines [111, 132] and an overexpression of adhesive molecules [111, 133]. Specifically, it has been documented that *Porphyromonas gingivalis* plays a main role in the promotion of LDL oxidation and plaque instability and rupture caused by metalloproteinase, as an initiating and promoting factor of peroxidation [111]. *Porphyromonas* is also involved in the progression of abdominal aortic aneurism [28, 134] and in inducing endothelial activation or dysfunction through a state of systemic inflammation with cytokines and metalloproteinase [111, 135–140]. In support of this evidence, there is a suggestive experiment demonstrating the effect of *Porphyromonas gingivalis* injection in mice fed with a hyperlipidic diet, where an increase of the atherosclerotic plaque thickness and of its lipid content has been observed [111, 123]. Similarly, a hypercholesterolemia-induced atherosclerotic process has been found following the injection of *Chlamydia pneumophila* [111, 141].

Finally, obesity, a growing problem in modern society, shares part of its pathogenesis and natural history with the host-colonizing microbiota, finding several meeting points with microbial metabolic influence. A characteristic *Firmicutes*/*Bacteroides* ratio has been discovered in obese patients with a surprising restoration of the normal proportion or lean-like proportion, once patients experienced a loss of weight [64, 92].

Several evidences are enlarging our knowledge and beliefs about the unavoidable influence of microbiota and its metabolome on our metabolic system, and the comprehension of this complex network is an absolute priority to introduce new therapeutic means and preventive solutions to slow or even stop the progression of atherosclerosis and its clinical manifestations, such as the strongly disabling diseases like the lower-limb PAD.

### 3.2. Restenosis after Percutaneous Angioplasty: The Possible Role of Microbiota

Angioplasty proves to be one of the most effective nonmedical treatments in diabetic PAD of the lower limbs, with the erroneous belief of gaining the vessel lumen enlargement in stented arteries rather than a simple balloon-angioplasty procedure. A recent study has demonstrated a loss in the lumen enlargement of the arteries treated by endovascular revascularization stenting caused by a neointimal hyperplasia that progressively reduces the vessel lumen and determines a restenosis of the treated vascular segment [80].

The effect of this hyperplastic phenomenon is the in-stent restenosis, a local process caused by hypercellularity and a low apoptosis rate [142].

This evidence deserves a notable scientific resonance because, according to collected data, the gut-related systemic inflammatory burden could be implicated in the neointimal hyperplasia, with a possible involvement in the in-stent microbe colonization as a further promoting factor [142].

In addition, different innate anatomic-functional characteristics of the arterial samples obtained from different body districts (e.g., the coronary artery and internal iliac artery) have been observed, suggesting an emergent necessity for new target-specific endovascular revascularization procedures and major vascular surgery for the PAD-affected population, rather than a translation of nonmedical treatments from the better-known coronary district to a totally different scenario as PAD [72, 143, 144].

Finally, more and more bacteria, correlated to the inflammation in the atherosclerotic process at the base of the restenosis mechanism, are being found (e.g., *Helicobacter pylori*, *Chlamydia pneumophila*). This evidence could introduce new therapeutic solutions against the in-stent restenosis, such as the addition of a microbe-specific antibiotic to the already used antiproliferation factors added to stent devices (e.g., Rapamycin) [28, 76] or the adjacent extravascular tissue antibiotic injection therapy with an expected prevention of neointimal hyperplasia and consequently in-stent restenosis [28, 71].

## 4. The Metabolome: From Waste to Biomarker

Since medical researchers have focused on the metabolic products of human multisliced colonizing microbiota film, a new interesting scenario has been proposed. The role of metabolites in host inflammatory process modulation and, consequentially, in atherosclerotic clinical manifestations as PAD has been defined to be much more essential and incisive than the producing microbe itself [145, 146].

An increasing number of studies enrich the knowledge about the gut metabolome by studying tryptophan (trp), kynurenine/tryptophan (kyn/trp) ratio, indole sulfonate, p-cresyl sulfonate (PCS), hippuric acid (HA), indole-3-carboxaldehyde (i3a), indole 3-proprionic (i3p), H_2_S, and phenylacetylglutamine and their influence on atherosclerotic phenomenon [147], like PAD in patients affected by a high grade of severe atherosclerosis, an end-stage disease characterized by an hemodynamic stenosis of carotid arteries aimed at an endarterectomy, disabling claudication, or critical limb ischemia undergoing an endovascular revascularization procedure or “demolitive” surgery with amputation in nonsolvable PAD [145]. Specifically, the tryptophan depletion determines an overactivation of the transduction signal of a stress pathway [145, 148] and an elevated value of the kyn/trp ratio is found in inflammatory statements, including infections with a proven positive relation to MACE. In addition, a low value of the kyn/trp ratio has been observed in germ-free mice with an interesting opposite tendency of this relation in case used for the first colonization of the same mice [145, 149, 150]. Data about the gut microbes' metabolic products are continuously developing with several examples of their effect on host homeostasis; for example, indole sulfonate has been observed to have an active role in VSMC dysfunction, vessel calcification, and thickening of arteries [145, 151]. It has been proven that PCS has a positive relation with cardiovascular death [145], while HA has an influencing role on postvascular surgery cardiac events and also a partially demonstrated positive correlation with ankle-brachial index (ABI), an accepted approximation of the high grade of atherosclerosis in PAD-affected patients [145].

In support of the demonstrative data showing the growing role of gut microbe metabolites in initiating and promoting the PAD process, a negative relation between indole, trp, i3p, and i3a and a high grade of carotid stenosis, disabling claudication, and critical limb ischemia (CLI) has been defined [145]. Meanwhile, higher baseline plasma concentrations of 3-hydroxyanthranilic acid and higher kyn/trp ratio have been traced in the advanced atherosclerosis group, mostly accepted in populations affected by CLI undergoing amputation of the lower limbs, clearly suggesting how a plasmatic concentration of trp greatly reduces the predisposition and risk of progression to an advanced phase of disease [145]. Similarly, increased levels of indole, i3p, i3a, and HA are detectable in patients with a higher ABI index, while on the other side, a negative correlation has been observed between the ABI index and high levels of 3-hydroxyanthranilic acid and high kyn/trp ratio [145].

Trimethylamine N-oxide (TMAO) deserves a particular description and focus. Recently, it has been defined as an independent risk factor for MACE [129, 152–157]. Trimethylamine (TMA) is a gut microbiota metabolite originating from the microbial metabolism of choline and found in many kinds of food as free choline or as a part of several compounds, such as betaine, L-carnitine derived from food [152, 153], and ergothioneine found in mushrooms, beans, and the liver and kidney of animals [152]. After the absorption from the gut lumen and once circulating in the bloodstream, TMA reaches the host liver where hepatic flavin monoxygenase produces TMAO [152]. The interest on this metabolite is derived from the observed positive relation between high levels of TMAO and markedly increased risk of atherosclerosis [129, 152–154, 158, 159]. The plasmatic levels of TMAO are influenced by diet with a higher plasma concentration in the case of elevated-fat-content diet, western diet, and red meat consumption [152, 153, 160–168]. On the other hand, a lower determination has been detected in patients affected by chronic kidney disease who respect a low-protein diet [152, 169]. The glomerular filtration rate acts like a determining factor of TMAO plasma concentration with an inverse proportion; therefore, there is an increase of TMAO levels in the case of a reduction of renal filtration ability and a restoration of healthy patient-like levels after kidney transplant [152].

It has been demonstrated that many human gut-colonizing bacteria are able to produce TMA increasing the TMAO plasma concentration (*Streptococcus sanguinis*, *Desulfovibrio alaskensis*, *Desulfovibrio desulfuricans*, *Acinetobacter*, *Serratia*, *Escherichia coli*, *Citrobacter*, *Klebsiella pneumoniae*, *Providencia*, *Shigella*, *Achiomobacter*, *Sporosorcine* that belongs to *Firmicutes phylum*, *Actinobacteria* [152, 170]). In contrast, bacteria belonging to *Bacteroidetes* are not capable of producing TMA [152, 166].

The characteristics of TMAO justify the importance of improving our knowledge about this metabolite. In fact, several responsibilities on creating an imbalance of host homeostasis have been described, such as endothelial dysfunction, oxidative-stress status promotion, overexpression of proinflammatory cytokines, and a positive relation with elevated inflammation biomarkers, incidence of T2DM, and chronic kidney disease [152].

In addition, TMAO appears essential for explaining part of the lipid balance and the increase of scavenger receptors (CD36 and scavenger receptor class A type 1 (SR-A1)), contributing to the rise of fat accumulation inside foam cells, a fundamental event of atherosclerotic plaque progress [129, 152, 171]. Flavin-containing monooxygenase 3 (FMO3) has been declared the most active in converting the liver enzyme of TMA in TMAO, and its activity is positively related to higher plasmatic levels of TMAO. FMO3 activation and the derived high levels of TMAO are strictly linked to an alteration of reverse cholesterol transport [152, 172], to facilitated hyperglycemia and hyperlipidemia (defined as increased levels of very-low-density lipoprotein- (VLDL-) and LDL-cholesterol) [152, 159, 172], to overexpression of TNF-*α*, IL6, CRP [162, 167], and insulin resistance [152, 159], and finally to the promotion of atherosclerosis [129, 152–154, 158, 159]. Therefore, TMAO has been demonstrated to be an independent influencing factor of the imbalance between the host metabolism and the inflammatory process. Moreover, it has been defined as transmissible atherosclerosis susceptibility factor [160].

Further studies have been conducted to understand clinical implications of TMAO. In fact, elevated plasmatic levels of this metabolite have a predictive role for the of 5-year all-cause mortality in stable patients affected by PAD [161]. This evidence could result essentially in the establishment of a new prognostic measurable blood marker to improve the stratification assessment of patients by detecting who deserves specific dietary supplementation or pharmacologic therapy [152, 153, 173–175].

Moreover, TMAO has been seen to be a quite precise predictive factor of the future risk of MACE and increased incidence of stroke, myocardial infarction, and death [129, 152–156, 160, 176–178]. This metabolite also has a positive correlation with Syntax scores I and II (angiographic grading tools to determine the complexity of coronary artery disease, the high values of which are related to cardiac mortality and MACE in patients undergoing multivessel and, specifically, unprotected left main percutaneous coronary intervention) even after adjustments for traditional risk factors [160].

## 5. Therapeutic Intervention

One of the first therapeutic proposals that have been suggested for application in the clinical practice is oral tolerance induction with self-antigens capable of reducing the inflammatory burden caused by the cross-mimicry phenomenon [72, 179, 180]. Another interesting proposal, which is even more successful, consists in dietary supplementation of immune-modulator, anti-inflammatory, and antiangiogenetic molecules containing food, such as catechin and epigallocatechine-3-gallate found in green tea. Surprisingly, a decrease of *Porphyromonas gingivalis*-related cytokine production in patients affected by periodontitis has been unveiled, giving discrete hopes in new therapeutic options to prevent and reduce the atherosclerotic process [181–185].

An additional possible new treatment could be fecal transplantation or the fragmented intestinal microbiota transplantation from lean healthy people that is described to be a hopeful treatment that reduces the insulin resistance and increases the butyrate producing microbiota [64, 186].

Furthermore, given the data regarding TMAO, the new therapeutic solutions could include a multifactorial reduction of TMAO plasmatic levels, for example, through targeting the gut microbiota TMAO producer [152] or the FMO3 enzymatic activity that reduces the conversion of TMA to TMAO [152]. An alternative option could be a change of dietary habits [152, 187], but it is not possible to reduce the carnitine and choline intake according to their nutritional importance [152]; therefore, it could be useful to encourage the consumption of marine fish which is rich in cardioprotective molecules such as *ω*3-polyunsaturated fatty acids (eicosapentaenoic acid (EPA) and docosahexaenoic acid (DHA)) that are implicated in the amelioration of impaired glycemic tolerance, in the reduction of adipose tissue-induced inflammation, in the reduction of monocyte chemoattractant protein-1 (MCP-1/CCL2), and in the increase of IL10 [152, 165].

Other possible solutions are the introduction of new effective prebiotics (all nondigestible food that stimulates the growth of beneficial bacteria) [152, 188] and probiotics (administrating specific bacterial strains such as *Lactobacillus paracasei*) [152, 189]. The use of antibiotics aimed at eliminating the TMAO producer microbes has also been proposed [152, 155]. Further therapeutic options are represented by the administration of an oral nonabsorbent binder to remove TMAO or its precursors [35, 170]; the inhibition of TMA precursors, for example, through 3,3-dimethyl-1-butanol (DMB) (contained in balsamic vinegar, red wine, extra virgin olive oil, and grape seed oil), that is an analogue of choline that competes and inhibits choline-TMA-lyase [152, 190]; and the inhibition of enzymes involved in TMA biosynthesis [152] using dietary supplements such as *Gynostemma pentaphyllum* [168] (an herbal product used in China to treat hyperlipidemia and obesity that is associated to a reduction of TMAO levels) or Gancao (the root of *Glycyrrhiza uralensis*) coadministered with a derivative of the *Aconitum carnichoelii* root [191]. Finally, it has been shown that enalapril is able to promote the renal excretion of TMAO [152, 175].

### 5.1. The Role of Antibiotics

The strong connection between the hosting organism and the colonizing microbiota has already been demonstrated and the scientific community continuously tries to collect new evidences about this crosstalk to find out new therapeutic ways and to manage the outcomes of the natural history of the disease.

The urgent necessity to fight a life-limiting disease such as PAD presents new challenges such as the achievement of cardioprotective therapeutic solutions through available sources. Nowadays, the use of available local modulators of gut microbiota, such as antibiotics and probiotics, has been demonstrated to be an effective protective factor for biologically different organs such as the cardiovascular system [192]. Mass spectrometry allows studying this revolutionary administration of exogenous influencing factors of microbes and the derived metabolome [192, 193] and permits comparing the metabolic paradigm/pattern between the examined case and the germ-free control [192, 194].

The oral administration of antibiotics and probiotics becomes the key to fully understand the role of human organism-colonizing microbes on our metabolic pathways. In fact, a direct modulation of the gut microbiome composition could indirectly determine an evident cardiovascular protective effect; on the other hand, the local injection of the same antibiotics in the coronary arterial circulation is associated to an ineffective cardioprotective outcome. In support of this evidence, during the trial of Lam et al., a group of mice premedicated with vancomycin alone or a combination of antibiotics (streptomycin, neomycin, bacitracin, and polymyxin B) showed a reduction of the necrotic myocardium after the induced coronary ischemia, compared to the control group treated with the same medication, directly injected in the coronary arterial circulation. Surprisingly, the administration of metabolites derived from phenylalanine, tryptophan, and tyrosine, at a sufficient concentration to restore the pretreatment serum levels, provokes the loss of the cardioprotective effect defined by the reduction of the size of the necrotized tissue area [192]. Notably, the used antibiotics are not absorbed and cannot reach the bloodstream, confirming the totally indirect cardioprotective mechanism. The direct effect on the gut microbe composition results in a reduction of *Clostridia* and a rise of *Bacilli* and *Proteobacteria* in the vancomycin-treated group, while it presents a reduction of *Bacilli* and no effect on *Clostridia* and *Proteobacteria* in the group treated with the mixture of antibiotics [192]. This evidence generates a new hypothesis: the cardioprotection mainly originates from the modification of the metabolome derived from the complex bacterial composition and interrelationship, rather than a specific *phylum*. It is possible that the interaction between circulating metabolites and cell surface receptors mutates the transduction signals of cellular survival pathways, leading to a worse cardiovascular outcome than the examined treated group, or they can be implied in the mitochondrial dysfunction worsening the evolution of the adverse event [192, 195].

Encouraging data have been derived from the comprehension of possible implicated cell signaling pathways, such as the Jak2 activity [192, 196]; the role of pyrazolopyrimidine on the Src family protein kinases [192, 197, 198]; the TGF*β*-mediated response [192, 199]; the effect of the fungal metabolite Wortmannin on mammalian target of rapamycin (m-TOR), a member of phosphatidylinositol-4,5-bisphosphate 3-kinase (PI-3) kinase superfamily; and other cellular transduction trails/paths worthy of further studies.

The established protective effect of orally administered antibiotics on remote organs such as the cardiovascular system demonstrated an effective reduction of the risk of restenosis or narrowing of the vessel treated with angioplasty, stenting, or bypass with graft. The production of short-chain fatty acids by the colonic bacteria fermentation of fibers taken with diet influences the function of VSMCs, the principle responsible for the vascular restenosis phenomenon [142]. The future will rely on a standardized antibiotic or probiotic administration as a treatment and a protective factor against the failure of the endovascular revascularization procedure that actually remains the main therapeutic option in PAD of the lower limbs.

Furthermore, the oral administration of vancomycin affects the gut microbiota composition of the host, at the cost of a minimal systemic absorbance, showing a relative decrease of Gram-positive bacteria belonging to *Firmicutes phylum* and the reduction of the plasmatic level of sodium butyrate [142, 200, 201] and presenting an increase of the *Bacteroidetes*/*Firmicutes* ratio and a rise of the Gram-negative *Proteobacteria* [142]. The result is a relative decrease of sodium butyrate Gram-positive producers, with a proof of an expected major neointimal hyperplasia observed in the vancomycin-treated group and an abolition of this same effect if the plasmatic concentration of sodium butyrate would have been restored by dietary supplementation, although with the concomitant administration of vancomycin. This confirms the antiproliferative and antimigratory properties of sodium butyrate on VSMC [142].

### 5.2. Probiotics in Diabetic PAD

Probiotics could gain an important role in the medical treatment of diabetic PAD. The rationale behind their use is based on the effect of an oral supplementation of microbes that shows a direct modification of the gut microbiota composition, which could be a further intervention against the dysbiosis found in this kind of patients [68].

Probiotics are revealing a complemental action with the antibiotic therapy in the selection of a protective combination of colonizing microbes. In fact, their systemic influence appears effective in ameliorating the lipid profile imbalance by reducing cholesterol plasmatic levels, increasing LDL-lipoprotein resistance against the oxidation, and inducing a decrease of the onset of insulin resistance in diabetic controls [202–204].

The contemporary administration of *ω*3 fatty acids has further empowered the effect of probiotics on host metabolism with an excellent result on lipid control, insulin resistance, and inflammatory response [202, 205–207].

Many alternatives have already been suggested as a possible probiotic therapy, in particular the probiotic VSL#3 (VSL Pharmaceuticals Inc., Fort Lauderdale, FL) which is notably interesting. It contains three strains of *Bifidobacteria* (*B. longum*, *B. infantis*, and *B. breve*), four strains of *Lactobacilli* (*L. acidophilus*, *L. paracasei*, *L. delbruceckii* subsp. *bulgarius*, and *L. plantarum*), and one strain of *Streptococcus salivaris* subsp. *termophilus*. The results observed during the use of VSL#3 confirm the usefulness of its introduction in the pharmacological therapy of PAD, with a further empowerment of the beneficial effect obtained by the addition of *ω*3 fatty acids as dietary supplementation. By the administration of probiotic VSL#3, interesting modulations on host metabolism have been described, such as the modest reduction of IL1, TNF-*α*, and IL6, the main responsible cytokines of the inflammatory atherosclerosis. Moreover, an increase of HDL cholesterol levels; a decrease of triglycerides, LDL, and VLDL lipoprotein levels; a decrease in fasting glycaemia and atherosclerotic index; and a marked modification of microbes in stool samples have been also demonstrated [202].

An effective risk factor control is at the base of the medical management of diabetic PAD, and the dietary supplementation of probiotics could appear as a new means to modify the natural history of this chronic, disabling, and progressive disease. It is necessary, however, to discover new combinations of supplementing microbes, focusing on their beneficial properties on systemic metabolism. It also important to respect the selection of the species contained in the probiotics, because there are strains of microbes that do not present an effective ability in the metabolic profile modulation, such as *Lactobacillus rhamnosus*, *Lactobacillus fermentum*, and *Lactobacillus acidophilus*, all of which have been observed ineffective in the improvement of serum lipid control [202, 208–210].

Moreover, the addition of prebiotics to the previously described probiotic treatment presented further interesting results, such as the reduction of plasmatic insulin with a consequential amelioration of the insulin resistance, a reduction of total and LDL cholesterol levels, and a reduction of triglycerides, accompanied by an elevation of HDL serum levels. In addition, an improvement of the inflammatory state has been described thanks to the decrease of CRP, IL1*β*, and TNF-*α* plasmatic concentrations. Moreover, in the symbiotic group, the one treated with probiotic and prebiotic supplementation, the count of *Lactobacilli* was higher, and the count of *Escherichia coli* and fecal coliform was lower [211].

**Figure 1:**
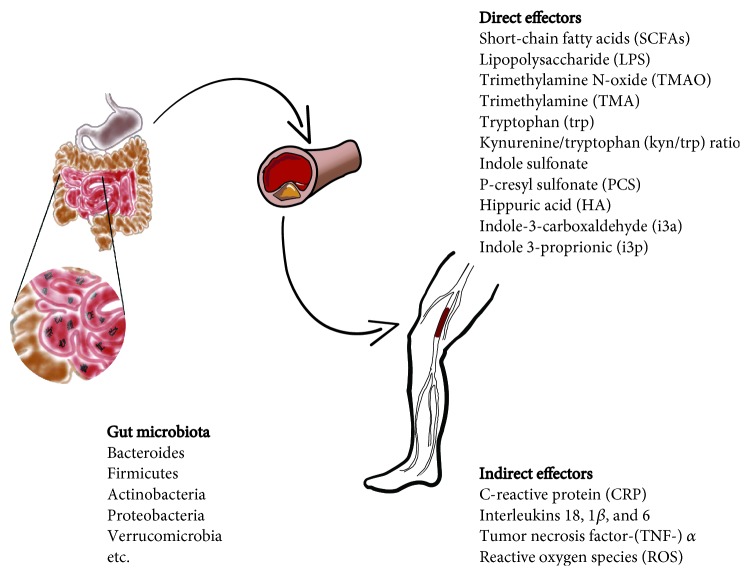
The different components of the intestinal microbiota (left) are able to worsen atherosclerosis at the base of the diabetic PAD by direct (top) and indirect (bottom) effectors.

A summary of the direct and indirect effectors involved in the connection between intestinal microbiota and PAD is reported in Figure 1.

## 6. Conclusions

In recent years, more and more evidences have documented the relationship between intestinal microbiota and diabetic PAD. The use of antibiotics is very frequent in patients affected by T2DM and PAD, since they often suffer from infected ulcers of the lower limbs. This could represent the first type of intervention: the choice of antibiotic therapies able to modulate, in one way or another, the intestinal microbiota represents an important objective. Furthermore, also the use of prebiotics and probiotics, useful for modifying the composition of the microbiota and the production of harmful metabolites, represents a further field of study. Finally, considering precision medicine, the study of a personalized therapy based on antibiotics, prebiotics, probiotics, and targeted diet could provide a new therapeutic instrument for the treatment of diabetic PAD.
